# Changes in serum creatinine in the first 24 hours after cardiac arrest indicate prognosis: an observational cohort study

**DOI:** 10.1186/cc8144

**Published:** 2009-10-29

**Authors:** Dietrich Hasper, Stephan von Haehling, Christian Storm, Achim Jörres, Joerg C Schefold

**Affiliations:** 1 Charité-Universitätsmedizin Berlin, Campus Virchow-Klinikum, Department of Nephrology and Medical Intensive Care, Augustenburger Platz 1, 13353 Berlin, Germany; 2 Charité-Universitätsmedizin Berlin, Campus Virchow-Klinikum, Department of Clinical Cardiology, Augustenburger Platz 1, 13353 Berlin, Germany

## Abstract

**Introduction:**

As patients after cardiac arrest suffer from the consequences of global ischemia reperfusion, we aimed to establish the incidence of acute kidney injury (AKI) in these patients, and to investigate its possible association to severe hypoxic brain damage.

**Methods:**

One hundred and seventy-one patients (135 male, mean age 61.6 +/- 15.0 years) after cardiac arrest were included in an observational cohort study. Serum creatinine was determined at admission and 24, 48 and 72 hours thereafter. Serum levels of neuron-specific enolase (NSE) were measured 72 hours after admission as a marker of hypoxic brain damage. Clinical outcome was assessed at intensive care unit (ICU) discharge using the Pittsburgh cerebral performance category (CPC).

**Results:**

AKI as defined by AKI Network criteria occurred in 49% of the study patients. Patients with an unfavourable prognosis (CPC 3-5) were affected significantly more frequently (*P *= 0.013). Whilst serum creatinine levels decreased in patients with good neurological outcome (CPC 1 or 2) over the ensuing 48 hours, it increased in patients with unfavourable outcome (CPC 3-5). ROC analysis identified DeltaCrea24 <-0.19 mg/dl as the value for prediction with the highest accuracy. The odds ratio for an unfavourable outcome was 3.81 (95% CI 1.98-7.33, *P *= 0.0001) in cases of unchanged or increased creatinine levels after 24 hours compared to those whose creatinine levels decreased during the first 24 hours. NSE levels were found to correlate with the change in serum creatinine in the first 24 hours both in simple and multivariate regression (both r = 0.24, *P *= 0.002).

**Conclusions:**

In this large cohort of patient after cardiac arrest, we found that AKI occurs in nearly 50% of patients when the new criteria are applied. Patients with unfavourable neurological outcome are affected more frequently. A significant association between the development of AKI and NSE levels indicating hypoxic brain damage was observed. Our data show that changes in serum creatinine may contribute to the prediction of outcome in patients with cardiac arrest. Whereas a decline in serum creatinine (> 0.2 mg/dL) in the first 24 hours after cardiac arrest indicates good prognosis, the risk of unfavourable outcome is markedly elevated in patients with constant or increasing serum creatinine.

## Introduction

Acute kidney injury (AKI) is a common and devastating problem in critically ill patients. Although sepsis is the most frequent cause of AKI in the intensive care setting, a number of other clinical conditions may induce renal failure [1]. Small changes in serum creatinine are associated with an increased mortality risk in hospitalised patients [2]. Following multiple and variable definitions of renal failure in the past, the Acute Kidney Injury Network has recently proposed uniform standards for diagnosing and classifying AKI [3]. This set of criteria has proven to be a valuable tool in various clinical situations [4-7]. Besides an improved definition of renal failure, much scientific effort has focused on the identification of the complex pathobiology of AKI in order to define new therapeutic targets. In recent years, animal models have mostly focused on renal ischemia and reperfusion (e.g. renal vascular cross clamp or high-dose norepinephrine infusion) [8]. Based on these studies renal ischemia/reperfusion is regarded as being a major contributor to the development of AKI in critically ill patients [9]. However, in the majority of patients it remains unknown whether AKI is caused by systemic versus renal hypoperfusion, circulating nephrotoxins, or additional insults.

Cardiac arrest may be considered as a model of systemic ischemia/reperfusion. Patients surviving cardiac arrest suffer from global ischemia/reperfusion affecting all end organs including the brain. Hypoxic encephalopathy is arguably the most important determinant of patient outcome in this setting, and it has been demonstrated previously that long-term prognosis depends more on the degree of hypoxic brain damage than on the underlying disease [10].

The extent of hypoxic brain damage can be estimated by measurement of serum levels of the enzyme neurone-specific enolase (NSE). This enzyme is a protein contained by neurons and is released into the circulation after neuronal cell damage. Peak serum levels reflect the amount of neuronal damage and correlate with clinical outcomes [11-13]. As a consequence, NSE serum levels may indicate the degree of hypoxic burden in patients surviving cardiac arrest.

Assuming that both the brain and kidney are sensitive to ischemia, hypoxic damage should affect both organs; however, few data to this end are presently available. We therefore set out to investigate the potential relation between hypoxic encephalopathy and AKI in patients after cardiac arrest. The new criteria defining AKI were applied to these patients and correlated to both NSE levels and short-term neurological outcome.

## Materials and methods

The study protocol was approved by the local ethics committee on human research. All data were collected within the normal daily intensive care routine in an anonymous fashion. The institutional review board therefore waived the need for informed patient consent. In a retrospective analysis, we identified a total of 195 patients who were admitted to the medical intensive care unit (ICU) of a tertiary care academic center after cardiac arrest between January 2003 and December 2007. In all patients, care was directed by critical care physicians based on standard operating procedures. Following our standard of care all patients received full ICU support over the first three days. Cardiac catheterization was performed as soon as possible when indicated. Patients admitted after December 2005 were treated with therapeutic hypothermia for 24 hours irrespective of the initial cardiac rhythm. According to our standard of treatment, neurological outcome was assessed after the third day using measures of clinical evaluation, NSE serum levels and somatosensory-evoked potentials when needed.

Seven patients died before the third day of ICU stay and were therefore excluded from further analysis. Another 12 patients were excluded due to incomplete data records and two patients because of pre-existing need for renal replacement therapy. Patients were excluded when pre-existing advanced renal disease was present. Advanced renal disease was defined as an estimated glomerular filtration rate less than 30 ml/min/1.73 m^2 ^at ICU admission (calculated using the simplified equation derived from the 'Modification of Diet in Renal Disease'(MDRD) study) [14]. Thus, three patients in Kidney Disease Outcome Quality Initiative stages 4 and 5, indicating severe and very severe renal failure, were excluded. The remaining 171 patients entered the analysis presented here. Blood samples for determination of serum creatinine levels were drawn immediately after ICU admission and every 24 hours thereafter. The difference between admission (i.e. baseline) serum creatinine and the values after 24 and 72 hours were calculated as ΔCrea24 and ΔCrea72.

AKI was defined by the criteria published by Mehta and colleagues [3] using the serum creatinine at admission as baseline value (Table 1). NSE serum levels were measured 72 hours after admission with an enzyme immunoassay (Elecsys 2010, Roche Diagnostics GmbH, Mannheim, Germany).

**Table 1:** Classification/staging system for acute kidney injury

Stage	Serum creatinine criteria	Urine output criteria
1	Increase in serum creatinine of more than or equal to 0.3 mg/dl (≥ 26.4 μmol/l) or increase to more than or equal to 150% to 200% (1.5 to 2-fold) from baseline	Less than 0.5 ml/kg per hour for more than 6 hours
2	Increase in serum creatinine to more than 200% to 300% (> 2 to 3-fold) from baseline	Less than 0.5 ml/kg per hour for more than 12 hours
3	Increase in serum creatinine to more than 300% (> 3-fold) from baseline (or serum creatinine of more than or equal to 4.0 mg/dl (≥ 354 μmol/l) with an acute increase of at least 0.5 mg/dl (44 μmol/l))	Less than 0.3 ml/kg per hour for 24 hours or anuria for 12 hours

Classification/staging system for acute kidney injury as provided by Mehta and colleagues [3]. Individuals who receive renal replacement therapy are considered to have met the criteria for stage 3 irrespective of the stage they are in at the time of renal replacement therapy.

Neurological outcome was assessed at the time of ICU discharge according to the Pittsburgh cerebral performance category (CPC) [15]. The classifying physician was blinded to the intention of the study. CPC 1 and 2 were classified as a favorable neurological outcome whereas CPC 3, 4 and 5 were regarded as an unfavorable outcome.

The software MedCalc^® ^9.3.2 (MedCalc Software, Mariakerke, Belgium) was used for statistical analysis. Continuous data are presented as median and 25 to 75% interquartile range (IQR) unless stated otherwise. Binary variables are presented as numbers and percentages. Mann-Whitney U testing was performed to compare continuous data, and Fisher's exact test was used to compare proportions. Simple and multivariable regression analyses were used as appropriate. Sensitivity and specificity of ΔCrea24 to predict outcomes were determined by analysis of receiver-operator characteristics (ROC) curves. The significance level was set at *P *< 0.05.

## Results

### Study population and neurological outcomes

Basic characteristics of the 171 cardiac arrest patients included in this study are presented in Table 2. With regards to neurological outcome, 69 patients had a favorable neurological outcome with either CPC 1 (n = 39, 22.8%) or CPC 2 (n = 30, 17.5%). Ten patients (5.8%) had moderate (CPC 3) and 24 patients (24.0%) severe neurological disability (CPC 4) at ICU discharge, and 68 patients (39.8%) died before ICU discharge (CPC 5). As a result of neurological assessment after the third ICU day, 87 patients (51%) had a do not resuscitate-order. Although those in the poor CPC group compared with the favorable CPC group were on average older, more likely to be female, and less likely to receive bystander cardiopulmonary resuscitation, these differences did not attain statistical significance. As expected, a favorable outcome was significantly associated with ventricular fibrillation as monitored as an initial rhythm, lower NSE serum levels and the application of therapeutic hypothermia.

**Table 2:** Baseline characteristics of study patients

	Study population (n = 171)	CPC 1 + 2 (n = 69)	CPC 3 + 4 + 5 (n = 102)	*P *value
Male gender	135 (78%)	58 (84%)	76 (74%)	0.18
Age (years)	63 (53-72)	60 (52-69)	65 (54-74)	0.13
OHCA	145 (86%)	62 (89%)	83 (81%)	0.08
Bystander CPR	46 (27%)	24 (35%)	22 (22%)	0.05
Cardiac cause of arrest	142 (83%)	62 (89%)	79 (78%)	0.06
VF as initial rhythm	111 (65%)	63 (91%)	48 (47%)	0.01
APACHE II score	28 (21-34)	29 (22-33)	27 (21-34)	0.59
Urine output (l/24 h)	2.0 (1.3-2.7)	2.0 (1.5-2.7)	1.9 (1.2-2.7)	0.18
Therapeutic hypothermia	98 (57%)	52 (75%)	46 (45%)	0.002
NSE after 72 hours (μg/l)	29.6 (18.5-80.9)	18.5 (12.5-23.7)	63 (29-203)	< 0.001
ICU length of stay (days)	13 (7-26)	15 (9-25)	13 (6-26)	0.13

Data are presented as medians (25th and 75th percentiles) or as absolute numbers (relative frequencies). APACHE: acute physiology and chronic health evaluation; CPC: cerebral performance category; CPR: cardiopulmonary resuscitation; ICU: intensive care unit; NSE: neuron-specific enolase; OHCA: out-of-hospital cardiac arrest; VF: ventricular fibrillation.

### Course of serum creatinine

In the overall study population a median serum creatinine at admission of 1.24 mg/dl (1.01 to 1.65 mg/dl) was measured. Over the ensuing two days, a significant drop of serum creatinine was observed with lowest values observed at ICU discharge (Table 3).

**Table 3:** Course of serum creatinine over time in patients after cardiac arrest

	Study population (n = 171)	CPC 1 + 2 (n = 69)	CPC 3 + 4 + 5 (n = 102)	*P *value
serum creatinine (mg/dl)				
at admission	1.24 (1.01-1.65)	1.20 (0.94-1.49)	1.32 (1.08-1.68)	0.039
after 24 hours	1.12 (0.74-1.87)	0.79 (0.60-1.49)	1.35 (0.96-2.06)	< 0.0001
after 72 hours	1.18 (0.79-2.23)	0.93 (0.67-1.50)	1.37 (0.92-2.51)	0.0174
at ICU discharge	0.86 (0.68-1.60)	0.78 (0.64-0.96)	1.05 (0.72-2.24)	0.0003
ΔCrea24	-0.12 (-0.35-0.30)	-0.25 (-0.51-0.02)	0.02 (-0.23-0.52)	< 0.0001
ΔCrea72	-0.01 (-0.33-0.68)	-0.13 (-0.38-0.24)	0.04 (-0.30-0.96)	0.026

Data are presented as medians (25th and 75th percentiles). The differences between patients with CPC 1 to 2 vs. CPC 3 to 5 were significant at every point of assessment. ΔCrea24: change in serum creatinine in the first 24 hours; ΔCrea72: change in serum creatinine in the first 72 hours; CPC: Cerebral Performance Category; ICU = intensive care unit.

A different pattern was observed when patients were stratified according to neurological outcome. In patients with unfavorable outcome (CPC categories 3 to 5, n = 102), serum creatinine was significantly higher at admission (1.32 vs. 1.20 mg/dl, *P *= 0.039) when compared with patients with favorable neurological outcome (n = 69). While serum creatinine levels on average decreased in patients with good neurological outcome in the following two days, they increased in average in patients with unfavorable outcomes.

### Frequency of AKI stages 0 to 3

A median urine output of 2000 mL (IQR 1300 to 2700 mL, range 0 to 10,080 mL) in the first 24 hours was found in the study population. There was no statistically significant difference between patients with good or unfavorable outcomes (*P *= 0.18, Table 2). Oliguria (urine output < 500 mL) was present in six patients with good outcome and in 11 with unfavorable outcome (*P *= 0.80). Renal replacement therapy was initiated in six patients with good outcome and in seven patients with unfavorable outcome (*P *= 0.88). Using serum creatinine levels at admission as baseline, AKI occurred more frequently in patients with unfavorable outcome. The difference compared with patients with good neurological outcome was statistically significant (*P *= 0.013, Table 4).

**Table 4:** Frequency of acute kidney injury stages 0 to 3

	Study population (n = 171)	CPC 1 + 2 (n = 69)	CPC 3 + 4 + 5 (n = 102)
AKI Stage 0	105 (61%)	50 (72.5%)	54 (52.9%)
AKI Stage 1	28 (16.3%)	9 (13%)	19 (18.6%)
AKI Stage 2	11 (6.4%)	3 (4.3%)	8 (7.8%)
AKI Stage 3	28 (16.3%)	7 (10.1%)	21 (20.6%)

Data are presented as absolute numbers (relative frequencies). AKI occurred significant more frequently in patients with unfavorable outcome (Chi-square test for trends, *P *= 0.013). AKI: acute kidney injury; CPC: cerebral performance category.

### NSE serum levels and univariate and multivariate regression

As expected, serum NSE values were significantly higher in patients with unfavorable outcomes (63 μg/L, IQR 29 to 203 μg/L, range 8.2 to 671 μg/L) compared with patients with good neurological outcome (18.5 μg/L, IQR 12.5 to 23.7 μg/L, range 4.8 to 58.3 μg/L, *P *< 0.001, Table 2).

Using simple regression we found that NSE levels correlated with ΔCrea24 (r = 0.24, *P *= 0.002), ΔCrea72 (r = 0.15, *P *= 0.049) and age (r = -0.17, *P *= 0.03), but not with Acute Physiology and Chronic Health Evaluation (APACHE)-II score, urine output and serum creatinine at admission (all *P *> 0.30).

NSE serum levels were analyzed with a multivariate regression model including gender, age, APACHE II-score at admission, urine output in the first 24 hours and change in serum creatinine in the first 24 hours (ΔCrea24) as independent factors. In this model, NSE levels were found to correlate with ΔCrea24 (r = 0.24, *P *= 0.0025) and age (r = -0.17, *P *= 0.048) independently of APACHE II-score (r = -0.014, *P *= 0.57), gender (r = 0.08, *P *= 0.21) and urine output (r = -0.07, *P *= 0.90). The multiple correlation coefficient was 0.31. The overall level of significance for the analysis of variance was *P *= 0.007.

A similar pattern was found when performing the analysis with outcome as the dependent variable. In this model, outcome was found to correlate with ΔCrea24 (r = 0.21, *P *= 0.0021) independently of APACHE II-score (r = -0.006, *P *= 0.21), gender (r = 0.18, *P *= 0.05), age (r = 0.003, *P *= 0.23) and urine output (r = -0.000009, *P *= 0.75). The multiple correlation coefficient was 0.29. The overall level of significance for the analysis of variance was *P *= 0.011.

### Risk stratification using ΔCrea24

The prognostic value of ΔCrea24 in predicting favorable neurological outcome was evaluated using ROC analyses. The area under the curve was calculated with 0.69 (95% confidence interval (CI) 0.62 to 0.76). The value for prediction of good outcome with the highest accuracy was ΔCrea24 less than -0.19 mg/dl. When this threshold was applied, good outcomes could be predicted with a sensitivity of 63% and a specificity of 71% (positive likelihood ratio (LR) 1.9, negative LR 0.4). Moreover, we found that the relative risk (RR) for unfavorable neurological outcome (CPC 3 to 5) was 2.1 (95% CI 1.5 to 3.0) in cases of unchanged or positive ΔCrea24 (*P *= 0.0001). When ΔCrea24 declined by more than 0.2 mg/dl, the RR for the occurrence of unfavorable neurological outcome was 0.46 (95% CI 0.32 to 0.68, *P *= 0.0001). The odds ratio was 3.81 (95% CI 1.98 to 7.33), *P *= 0.0001 or 0.27 (95% CI 0.14 to 0.51, *P *= 0.0001), respectively. For interval LRs for ΔCrea24, please refer to Table 5.

**Table 5:** Interval likelihood ratios for ΔCrea24.

ΔCrea24 (mg/dl)	CPC 1 + 2 (n = 69)	CPC 3+4+5 (n = 102)	Likelihood ratio	95% confidence interval
<-0.4	27	10	0.251	0.130 to 0.484
-0.4--0.2	15	20	0.902	0.497 to 1.636
-0.2-0.0	8	19	1.607	0.746 to 3.461
0.0-0.2	8	17	1.437	0.657 to 3.145
0.2-0.4	3	8	1.804	0.496 to 6.562
> 0.4	8	28	2.368	1.148 to 4.883

Interval likelihood ratios with 95% confidence interval for ΔCrea24. The number of patients with unfavorable vs. favorable outcome is given for respective ΔCrea24 intervals. ΔCrea24: change in serum creatinine in the first 24 hours; CPC: Cerebral Performance Category.

## Discussion

We demonstrate that AKI is common in patients after cardiac arrest when the new AKI criteria are applied. Patients with unfavorable neurological outcome are affected significantly more frequently. Furthermore, we found a direct significant association between AKI and serum levels of NSE as a marker of hypoxic brain damage.

AKI is a known complication after cardiac arrest although different definitions of 'renal failure' in the past have made comparisons difficult [16]. In a recent investigation some pre-arrest factors including history of hypertension, chronic heart failure and chronic renal insufficiency could be identified as risk factors for renal failure after cardiac arrest and an association between acute renal failure and epinephrine dosage during cardiopulmonary resuscitation was found [17]. This may indicate that the extent of hypoxia/ischemia may also play a role in the development of AKI. In fact, acute renal failure could be induced by cardiac arrest in a mouse model [18]. This finding is in line with our data suggesting an association between hypoxia and the development of AKI after cardiac arrest. Furthermore, our data indicate that early changes in serum creatinine might help to predict outcome in these patients: while a decline of serum creatinine levels of 0.2 mg/dl or more in the first 24 hours after cardiac arrest may indicate good prognosis, constant or even increasing levels seem to predict unfavorable outcome.

A number of limitations to our analysis require careful consideration. First, our data were obtained in a single-center cohort and thus require validation studies before clinical application. Second, the detection of differences in creatinine levels of 0.2 mg/dl is sophisticated with regard to the precision of the test. Furthermore, data about kidney function prior to cardiac arrest were not available. This may be an important point because our data suggest a temporary rise in serum creatinine during the first hours after successful cardio-pulmonary resuscitation. Although an early rise in serum creatinine following cardiac arrest was also found in previous observations, the reason for this phenomenon remains unknown [19]. On the whole, it is not clear if such early changes in serum creatinine indeed represent real alterations in glomerular filtration rate. Thus, serum creatinine at admission is not a reliable measure for chronic kidney function in these patients. As we have used the serum creatinine at admission as the baseline for defining AKI, the incidence of acute kidney disease may be underestimated in our cohort.

Moreover, creatine release from skeletal muscles during cardiopulmonary resuscitation may theoretically influence the course of serum creatinine levels. Although we are unable to rule out an effect of muscular release of creatine with certainty, serum creatine kinase levels were not found to correlate with serum creatinine levels or with changes in serum creatinine levels at baseline and over time (*P *> 0.5 for all comparisons). Moreover, serum creatine kinase was not found to discriminate between favorable and unfavorable outcome (data not shown). Kidney function may be also affected by treatment with therapeutic hypothermia. Although recent investigations did not detect differences in the incidence of acute renal failure under hypothermia, transient effects on renal function cannot be fully excluded [20].

Concerning neurological outcome, we only present CPC scores at ICU discharge. Although some evidence indicates that there are only minor changes regarding neurological outcome after ICU discharge [21], long-term follow up may provide more insight into this important endpoint. Moreover, one should keep in mind that classification as CPC 5 may reflect two different clinical situations: Patients dying in a comatose state after therapy withdrawal and patients dying from other complications after regaining consciousness. Nevertheless, although the neurological situation seems completely different, from the patient's point of view CPC 5 is an important outcome variable independent of the cause of death. In addition, there is good evidence that the majority of patients after cardiac arrest die after therapy withdrawal [22].

Importantly, our data should not be used to predict outcome in patients after cardiac arrest. Obviously, when predicting neurological outcome one should focus on the brain, not the kidney, and reliable multimodal approaches are available for this purpose [23]. Nevertheless, the demonstrated relation between the kidney and the brain may help to identify patients at a high risk of an unfavorable outcome. Theoretically, this may have implications for ICU care in the future. In patients with sepsis, convincing data demonstrate that early identification and therapy using an early goal-directed therapeutic approach with fluids and vasopressor support improves organ function and outcome [24]. Although somewhat speculative, one might argue that these rather simple approaches may also be effective in patients after cardiac arrest via improvement of both cerebral and kidney function.

Moreover, there may be another conclusion which may be drawn from our data. Nearly half of the patients with severe hypoxic brain damage after cardiac arrest did not develop AKI despite profound global ischemia. This result is in marked contrast to the situation typically found in severe shock and multiple-organ failure where acute renal failure is a common condition but relevant encephalopathy a comparably rare event. In this light, our findings support the hypothesis that 'simple' hypoperfusion may be only one piece in the puzzle of the complex pathophysiology of AKI. In fact, mounting evidence from animal models indicate that AKI may develop without renal ischemia in sepsis [25-27]. As a consequence, critical care physicians should be once more careful when extrapolating results obtained from animal models to the clinical situation of our patients. One might speculate that eventually we will need to differentiate 'high flow' from 'low flow' AKI and accordingly apply different treatment strategies in the future.

## Conclusions

In summary, we demonstrate that AKI occurs in nearly 50% of patients after cardiac arrest when the new AKI criteria are applied. Patients with unfavorable neurological outcome are affected more frequently. Furthermore, we found a significant association between AKI and serum levels of NSE as a marker of hypoxic brain damage. Our data indicate that changes in serum creatinine might be an early predictor of outcome in these patients and that a decrease of serum creatinine in the first 24 hours of more than 0.2 mg/dl may be a sign of good prognosis, whereas constant or even increasing serum creatinine levels indicate unfavorable outcomes.

## Key messages

• AKI is frequent after cardiac arrest - approximately 50% of cardiac arrest patients may be affected.

• Development of AKI is associated with unfavorable neurological outcome and increased mortality in patients after cardiac arrest.

• Whereas a decline in serum creatinine levels in the first 24 hours may indicate favorable prognosis, constantly elevated or even further increased serum creatinine levels indicate an unfavorable outcome after cardiac arrest.

## Abbreviations

ΔCrea24: change in serum creatinine in the first 24 hours; ΔCrea72: change in serum creatinine in the first 72 hours; AKI: acute kidney injury; APACHE: Acute Physiology and Chronic Health Evaluation; CI: confidence interval; CPC: Cerebral Performance Category; ICU: intensive care unit; IQR: interquartile range; LR: likelihood ratio; NSE: neuron-specific enolase; ROC: receiver-operator characteristics; RR: relative risk.

## Competing interests

The authors declare that they have no competing interests.

## Authors' contributions

DH, CS, SvH and JCS designed and supervised the study from data acquisition to data analysis. AJ participated in the design of the study, revised the manuscript for important intellectual content and helped to draft the manuscript. All authors have read and approved the final version of the manuscript.
